# Interaction of the Gut Microbiota and the Enteric Nervous System: Implications in Gastrointestinal Disorders Associated With Autism Spectrum Disorder

**DOI:** 10.1002/aur.70328

**Published:** 2026-07-26

**Authors:** Baptiste Ganachaud, María José Mendoza‐León, Justine Marchix, Alonso Fernandez Tello, Catherine Le Berre Scoul, Ma Cecilia Opazo, Michel Neunlist, Claudia A. Riedel, Martial Caillaud, Hélène Boudin

**Affiliations:** ^1^ INSERM UMR 1235, TENS‐The Enteric Nervous System in Gut and Brain Disorders, IMAD Nantes Université Nantes France; ^2^ Millennium Institute on Immunology and Immunotherapy Santiago Chile; ^3^ Centro de Investigación de Resiliencia a Pandemias, Facultad de Ciencias de la Vida Universidad Andrés Bello Santiago Chile; ^4^ Centro de Investigación en Ciencias Biológicas y Químicas, Instituto de Ciencias Naturales, Facultad de Medicina Veterinaria y Agronomía Universidad de las Américas Santiago Chile

**Keywords:** autism, bacterial metabolites, enteric nervous system, enteric neuron, gut‐brain axis, microbiota

## Abstract

Autism spectrum disorder (ASD) is a neurodevelopmental disorder characterized by impairments in social interaction, restricted interests, and repetitive behaviors. In addition to these core behavioral symptoms, gastrointestinal (GI) disorders are frequently reported, ranging from severe constipation to diarrhea. The links between ASD and gut dysfunction are further supported by alterations in gut microbiota composition and in bacteria‐derived metabolites. As the intrinsic nervous system of the digestive tract, the enteric nervous system (ENS) plays a central role in gut physiology by exerting neuronal control on several gut functions, including motility. Located at the interface between the gut microbiota and intestinal function, the ENS may play a key role in the GI symptoms associated with ASD, as demonstrated in several mouse models. This review aims to summarize the interconnected alterations of the gut microbiota and the ENS in ASD, with a particular focus on the microbiota‐derived mediators that are altered in ASD, among patients and animal models, and that may affect ENS development and function. By highlighting these interactions, this review seeks to provide new insights on the digestive pathophysiology of ASD, which may also contribute to the severity of behavioral symptoms.

## 1 Introduction

Autism spectrum disorder (ASD) is a neurodevelopmental disorder that emerges in early childhood. According to the Diagnostic and Statistical Manual of Mental Disorders version 5 (DSM‐V) (American Psychiatric Association and Gunderson 2017), ASD is characterized by altered social interaction and communication in association with repetitive behavior and restricted interests. The global prevalence of autism is constantly increasing and is estimated to be around 1% in the general populations (Zeidan et al. 2022). The etiology of ASD involves genetic and environmental factors (Lai et al. 2014). With regard to genetic factors, more than 100 genes have been implicated in the risk of ASD. Most of these genes encode synaptic scaffolding proteins, receptors, cell adhesion molecules, or proteins involved in chromatin remodeling, transcription, protein synthesis and degradation, as well as actin cytoskeleton dynamics (Bourgeron 2015; Satterstrom et al. 2020). In addition, exposure to environmental factors during the in utero period and childhood, such as advanced maternal and paternal age, metabolic conditions, thyroid hormone imbalance, hypertension and exposure to different toxic substances, pollutants, pesticides as well as bacterial or viral infections, has also been associated with an increased incidence of ASD (Bölte et al. 2019; Elbedour et al. 2025; Love et al. 2024; Rossignol et al. 2014; Rotem et al. 2020). Despite the paucity of direct evidence, converging findings from human and animal studies link ASD to defects in brain connectivity and synaptic function (Holiga et al. 2019; Matuskey 2025).

ASD is also associated with a wide range of comorbidities, including neurological conditions such as epilepsy, sleep disorders, anxiety, and obsessive‐compulsive disorders (Lai et al. 2014; Lord et al. 2018). In addition, people with ASD have a higher prevalence of gastrointestinal (GI) disorders compared with neurotypical population (McElhanon et al. 2014). It is estimated that 50%–70% of people with ASD experience GI symptoms, including constipation, diarrhea, bloating and abdominal pain (Lai et al. 2014). Interestingly, several studies have reported correlations between the severity of GI disturbance and behavioral symptoms (Adams et al. 2011; Chakraborty et al. 2021), suggesting shared underlying mechanisms in gut and brain dysfunctions. The connection between ASD and gut dysfunctions is further supported by evidence of gut microbiota dysbiosis and alterations in bacteria‐derived metabolites (Kang et al. 2018; Liu, Li, Wu, et al. 2019; Morton et al. 2023). Another factor implicated in GI disorders in ASD is the enteric nervous system (ENS). As an intrinsic component of the digestive tract, the ENS plays a key role in gut physiology by exerting neuronal control on gut permeability and motility. Positioned at the interface between gut microbiota and intestinal function, the ENS may be a key actor in the GI symptoms associated with ASD, as demonstrated in several ASD mouse models. Moreover, the ENS may also represent a gateway to the brain via the vagus nerve, which mediates bidirectional communication between the gut/ENS and the brainstem through 80% and 20% of afferent and efferent fibers, respectively. Afferent fibers sense the luminal content of the gut and transmit signals to the brain, whereas efferent fibers control gut motility and secretory functions through direct interactions with enteric neurons (Jiang et al. 2024; Powley 2021). Interestingly, reduced vagal nerve activity has been reported in children with ASD (Matsushima et al. 2016), which could impair communication between the ENS and the brain, thereby potentially contributing to both gastrointestinal and behavioral disorders. In addition, the ENS may influence other body systems through connections with the sympathetic nervous system via viscerofugal neurons (Hibberd et al. 2020). As alterations in the sympathetic pathway have been reported in some individuals with ASD (Zadok et al. 2024), it is important to consider the potential role of the ENS in these disturbances.

Recent reviews have described the impact of autism‐associated genetic factors modeled in mouse models on the ENS structure and functions (Lee et al. 2020; Wang, Tang, et al. 2023). However, a comprehensive analysis linking the ENS with bacterial metabolites produced by the gut microbiota, which show altered levels in autism, is still needed to better understand the potential interactions between the gut microbiota, the ENS, and the gastrointestinal disorders associated with autism.

To conduct this study, we based our work on a targeted literature search conducted in PubMed and Web of Science to identify studies examining the relationships between ASD, the gut microbiota, and the enteric nervous system. Search terms included combinations of “autism spectrum disorder,” “autism,” “gut microbiota,” “microbiome,” “bacterial metabolites,” “enteric nervous system,” “enteric neurons,” “enteric glial cell,” “gut–brain axis,” and “gastrointestinal disorders.” Priority was given to peer‐reviewed articles published in English, with an emphasis on recent studies and seminal papers providing mechanistic insights. Both clinical and preclinical studies, including animal models, were considered to capture complementary perspectives on ENS development and function in the context of microbiota alterations. Studies were selected based on their relevance to the interplay between microbial composition, microbiota‐derived metabolites, and ENS regulation, while efforts were made to include findings that both support and challenge current hypotheses.

Through this literature analysis, this review aims to synthesize current knowledge on the interconnected alterations of the gut microbiota and the ENS, with a particular focus on the microbiota‐derived mediators that are altered in ASD in both patients and animal models and that may affect ENS development and function. In the vast majority of studies, the links between the ENS and ASD are primarily based on analyses of enteric neurons, while only a limited number of studies have investigated enteric glial cells in the context of ASD. Therefore, this review mainly focuses on studies involving enteric neurons.

## 2 The Enteric Nervous System (ENS)

### 2.1 Presentation of the Enteric Nervous System (ENS)

**FIGURE 1:**
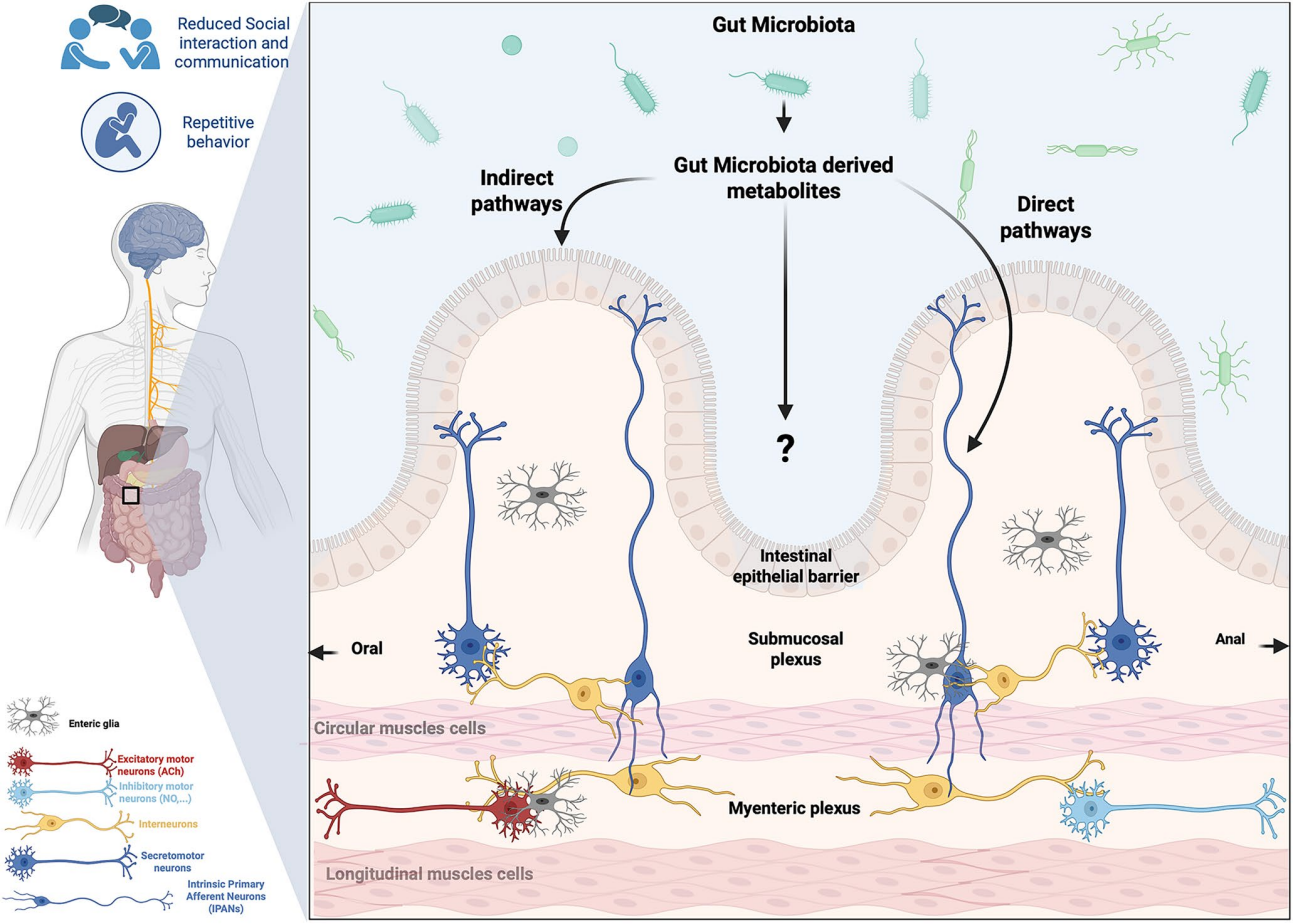
Gut microbiota–enteric nervous system interactions and their potential involvement in autism spectrum disorder (ASD). The microbiota produces numerous metabolites that impact intestinal function and neural communication. These microbial metabolites may influence the ENS directly or indirectly, possibly by impacting the intestinal epithelial barrier.

The ENS is a subdivision of the autonomic nervous system, itself part of the peripheral nervous system, distributed throughout the digestive tract within the gut wall. The development of the ENS has been extensively reviewed elsewhere (Avetisyan et al. 2015; Hao et al. 2013; Kang et al. 2021). Here, we provide an updated summary of the current knowledge based mainly on studies conducted in mice. The ENS development is initiated at embryonic stage (E)9.5 with the migration of vagal enteric neural crest cells (ENCCs) from the neural crest of the ectodermal embryonic layer into the foregut, along a rostro‐caudal direction (Kang et al. 2021). During migration, ENCCs proliferate and differentiate into enteric neurons and glia upstream the migratory wavefront at the level of the prospective myenteric plexus (MP). As ENCCs cluster and form ganglia in the myenteric layer, neurites extend, and a subset of progenitors in the small intestine begins to project radially toward the mucosa, giving rise to the submucosal plexus (SMP) by E15.5. Gut colonization by ENCCs is completed by E15.5. ENS maturation continues postnatally by the increase of axonal length and arborization, along with axonal fasciculation and synapse formation (Parathan et al. 2020). These processes of migration, proliferation, and differentiation are tightly regulated by a complex network of transcription factors and signaling molecules present in the microenvironment. Together, they result in the formation of the ENS as two distinct ganglionated neuronal plexuses, the MP and the SMP (Figure 1). The MP is located between the longitudinal and circular muscle layers, while the SMP is located beneath the mucosa. The MP primarily regulates gut motility through the coordination of smooth muscle contractions, that drive luminal content propulsion (Spencer and Hu 2020). Reflex activity is initiated by intrinsic primary afferent neurons (IPANs), that detect mechanical stimuli and luminal chemical signals (Furness 2012). IPANs express various neurotransmitters including acetylcholine (Ach), CGRP, calbindin and tachikinin (Rao and Gershon 2016), and form synapses with both ascending and descending interneurons within the myenteric plexus (Figure 1). The ascending interneurons regulate excitatory motor neuron activity through the release of ACh and enkephalin, while descending interneurons control inhibitory motor neurons through acetylcholine and serotonin (5‐HT). Motoneurons producing Ach and substance P induce oral contraction of smooth muscle, while those producing nitric oxide (NO) and vasoactive intestinal peptide (VIP) induce anal relaxation. In addition to motility control, the ENS regulates intestinal permeability via secretomotor neurons located in the SMP (Furness 2012). Within this plexus, vasomotor neurons also contribute to the regulation of the vasodilatation of blood vessels. Both secretomotor and vasomotor neurons express ACh and VIP. While the ENS is capable of operating independently of the central nervous system (CNS), the gut remains anatomically and functionally connected to the CNS through vagal, sympathetic and pelvic pathways (Furness 2012). In particular, the vagus nerve supports bidirectional communication between the two organs, with approximately 90% of its fibers being afferent (Rao and Gershon 2016). After birth, the GI tract is colonized by the microbiota (Obata and Pachnis 2016) which contribute to ENS development and function. Gut microbiota is essential for the maintenance of ENS integrity by regulating enteric neuronal survival and promoting neurogenesis (Vicentini et al. 2021). Studies with germ‐free mice have shown that, as early as postnatal day 3, the ENS exhibits a reduced neuronal density and an increased proportion of nitrergic neurons, identified by the expression of the neuronal nitric oxide synthase (nNOS), the enzyme responsible for NO synthesis, compared with conventionally colonized mice (Collins et al. 2014), suggesting a role of the gut microbiota in postnatal neuronal plasticity. In adult mice, gut microbiota has been shown to influence ENS structure in a serotonin (5‐HT)‐dependent manner, leading to alterations in intestinal transit (De Vadder et al. 2018). One of the mechanisms underlying the effects of the gut microbiota on the ENS involves the regulation of enteric neuronal transcriptional profiles, notably through the induction of the aryl hydrocarbon receptor (AhR) expression in enteric neurons, thereby modulating the neurogenic programs controlling gut motility (Obata et al. 2020). The gut microbiota also affects enteric glial cells by regulating their initial colonization and homeostasis within the intestinal mucosa and the myenteric plexus of the ileum (Vicentini et al. 2021; Kabouridis et al. 2015). Overall, these studies highlight the significant role of microbial colonization in shaping the development and function of the ENS.

### 2.2 ENS Alterations in Gastrointestinal Disorders Associated with ASD in Humans

Consistent with the central role of the ENS in regulating GI physiology, structural and functional alterations of the ENS have been linked to several GI disorders. These disturbances may arise from genetic mutations affecting ENS development, as well as from changes in neurochemical coding, neuronal density, or connectivity, ultimately contributing to motility disorders that impair GI transit. For a comprehensive overview of ENS involvement in GI pathologies, readers are referred to recent reviews (Holland et al. 2021; Niesler et al. 2021).

GI disorders are among the most prevalent comorbidities in individuals with ASD, occurring 4.4 times more frequently in autistic children compared to neurotypical individuals. Constipation is the most frequently reported symptom, followed by diarrhea and abdominal pain (McElhanon et al. 2014; Madra et al. 2021). Despite this high prevalence, no histopathological studies to date have examined ENS alterations in intestinal biopsies from ASD patients. Nevertheless, parallels can be drawn between GI symptoms observed in ASD and those reported in other digestive disorders for which ENS alterations have been documented. For instance, in patients with slow‐transit constipation, colonic biopsies have revealed ENS abnormalities such as hypoganglionosis, characterized by reduced ganglion size and density in the myenteric and submucosal plexuses (Wedel et al. 2002). In addition, changes in the neurochemical phenotype of enteric neurons have been described in individuals with chronic constipation, including an increased density of nitrergic neurons alongside a decreased density of VIPergic and cholinergic neurons in the colon (Cortesini et al. 1995; Tian et al. 2025). Other symptoms commonly reported in children with ASD, such as diarrhea, abdominal pain, and bloating (Leader et al. 2022), currently lack direct evidence linking them to ENS alterations in humans, even beyond the context of autism. Notably, many of these symptoms are also characteristic of disorders such as irritable bowel syndrome (IBS), which presents with overlapping clinical features including abdominal pain with diarrhea or constipation, or a combination thereof (Grundmann and Yoon 2010). Importantly, more than half of autistic children may exhibit IBS‐like GI comorbidities (Penzol et al. 2019; Sanctuary et al. 2019). Genetic studies further support this association, revealing a partially shared genetic architecture between ASD and IBS, which may partly explain their frequent co‐occurrence (Li et al. 2024). In IBS, intestinal mucosal biopsies have shown increased nerve fiber density and enhanced neurite outgrowth (Dothel et al. 2015), although the precise contribution of the ENS in these changes remains unconfirmed. Considering that functional constipation and IBS with constipation are associated with dysbiosis of the gut microbiota (Ohkusa et al. 2019) and that colonic transit time is a key determinant of the gut microbiota composition (Procházková et al. 2023), it is possible that chronic constipation alters microbial community and secondarily affects the ENS.

Nevertheless, the structure and integrity of the ENS have yet to be directly investigated in ASD patients, since most of the available evidence currently derives from genetic or environmental animal models of ASD, as discussed below.

### 2.3 Alteration of ENS in Animal Model of ASD

Numerous animal models of ASD have consistently demonstrated alterations in the brain; however, several studies have also reported changes in other systems, including the ENS. Major deficits in the ENS observed in both genetic and environmental models of ASD have been recently reviewed (Wang, Tang, et al. 2023). Herein, we provide an updated overview by incorporating the most recent findings on ENS alterations in animal models of ASD.

Animal models have become essential tools for investigating the pathophysiological, behavioral, and brain alterations associated with ASD. Given the high prevalence of GI disorders in ASD, an increasing number of studies now also focus on identifying the pathophysiological mechanisms underlying digestive tract dysfunctions, with particular attention to the ENS.

Genetic animal models of ASD largely rely on transgenic mice carrying mutations in ASD susceptibility genes identified in individuals with ASD (Silverman et al. 2010). These genes primarily encode proteins involved in neural circuit development, particularly those implicated in synapse formation and function (Satterstrom et al. 2020). Initially, these models were studied for their effects on the central nervous system through behavioral assessments and assessments of brain connectivity. However, the high rate of GI comorbidity in ASD, potentially linked to ENS abnormalities, has prompted renewed interest in evaluating their impact on digestive function and ENS structure.

Several ASD‐associated gene variants have been shown to disrupt the migration of enteric neuronal progenitors during ENS development. A recent study highlighted key genes involved in these processes, including *SYNGAP1* and *SCN2A*, which encode proteins critical for neurotransmission (McCluskey et al. 2025), *DYRK1A*, which encodes a kinase, and chromatin‐regulating genes, such as *CHD2* and *CHD8*. Theses genetic alterations impair neural progenitor migration in vivo, potentially contributing to gut dysmotility commonly observed in individuals with ASD (McCluskey et al. 2025).

In a zebrafish model, *CHD8* mutation was associated with a reduced number of enteric neurons, further supporting the link between ASD‐related genetic alterations and ENS abnormalities (Bernier et al. 2014). Changes in the density of enteric neurons have also been reported in mouse models targeting synapse‐related genes. Mice carrying a mutation in the neuroligin 3 gene, *Nlgn3* R451C, display an increased density of nitrergic neurons in the jejunum compared to wild‐type mice (Table 1) (Hosie et al. 2019), although *Nlgn3* knockout shows no difference in neuron number across gut regions (Leembruggen et al. 2020). Despite these differences in ENS phenotype, both models showed accelerated small‐intestinal transit. Also, mice lacking *Shank3B*, a synaptic scaffolding protein important for synaptic transmission and linked to ASD, show increased density of enteric neurons in the colonic myenteric plexus (Eberly et al. 2025). In contrast, a zebrafish model carrying C‐terminal frameshift mutations in *Shank3a/b* showed no modification in the number of enteric neurons. Nevertheless, both models display digestive dysmotility, characterized by reduced peristaltic contractions and prolonged transit time (Table 1) (Eberly et al. 2025; James et al. 2019).

**TABLE 1:** Enteric nervous system alterations in genetic and environmental ASD models.

	Type of gene or environmental factor	Type of mutation or mode of exposure	Animal model or samples	Bacterial metabolites composition alterations	ENS alterations/gastrointestinal dysfunctions	Publications
Genetic models	Neuroligin3	R451C	Mouse	NI	Increased jejunal neuronal density, particularly nitrergic neurons ➔ Small intestinal motility.	(Hosie et al. 2019)
		KO	Mouse	NI	No changes in enteric neuron populations but increased colonic diameter and faster colonic motility.	(Leembruggen et al. 2020)
	SERT	KO	Mouse	NI	Increase number of enteric neurons ➔ ENS hyperplasia.	(Margolis et al. 2016)
		Ala56 variants	Mouse	NI	Decresed number of enteric neurons ➔ ENS hypoplasia.	(Margolis et al. 2016)
	SHANK3	SHANK3−/− KO	Mouse	Decreased cecal levels of SCFA: Acetate	Increased myenteric plexus density and a higher number of HuC/D expressing neurons in myenteric ganglia	(Eberly et al. 2025; Osman et al. 2023)
		SHANK3a/b +/−	Zebrafish	NI	Reduced peristaltic contractions and Prolonged transit times in digestive tract	(James et al. 2019)
	CHD8	CHD8+/−	Mouse	Reduced levels of fecal tryptophan	Reduced ENCC migration 50% reduction of enteric neurons.	(Bernier et al. 2014; Yu et al. 2022)
	TPH2	KO	Mouse	NI	Decrease of myenteric neuronal density and proportions of dopaminergic and GABAergic neurons	(Li et al. 2011)
	Cntnpa2	Cntnpa2−/−	Mouse	Reduced BH4 metabolite in feces	Affects sensory neuron excitability and colonic motility.	(Robinson et al. 2023; Buffington et al. 2021)
	BTBR	BTBR T+ Itpr3^tf/J^	Mouse	Deficient bile acid and tryptophan metabolism in the intestine	Reduced neuron density in myenteric plexus.	(Golubeva et al. 2017)
Environmental models	Maternal immune activation (MIA)	Maternal gestational exposure to poly(I:C)	Mouse	Decreased serum level of indole pyruvate	MIA mice showed a deficiency in CGRP fiber innervation in the colon.	(Li et al. 2023; Hsiao et al. 2013)
	Valproic acid	Maternal gestational exposure to valproic acid	Mouse	Steroid hormones biosynthesis enrichment	Decreased expression of PGP9.5 in enteric neurons.	(Cheng et al. 2020; Gu et al. 2022)

Abbreviation: NI: not identified.

Another mouse model of ASD lacking contactin‐associated protein‐like 2 (*CNTNAP2*), a gene encoding a cell‐adhesion molecule important for neuronal circuit development, and mutated in some individuals with ASD (D'Onofrio et al. 2023), showed no changes in enteric neuron or glial cell numbers. However, *CNTNAP2* deletion affected colonic motility, reducing transit time in empty colons while accelerating transit in the presence of luminal stimuli (Table 1) (Robinson et al. 2023).

Margolis et al. (2016) have described ENS alterations in animal models carrying either a mutation at alanine 56 or a deletion of the serotonin (5‐HT) transporter *SERT* (Table 1). Dysregulation of 5‐HT signaling has also been linked to ASD. In particular, deletion of tryptophan hydroxylase 2 (*TPH2*), the rate‐limiting enzyme in 5‐HT synthesis, has been associated with autism‐related behavioral phenotypes, including altered patterns of social behavior and vocalization patterns in rats (Golebiowska et al. 2025). In *TPH2* knockout mice, ENS abnormalities include reduced myenteric neuronal density and altered proportions of dopaminergic and GABAergic neurons, which are associated with decreased intestinal transit and accelerated gastric emptying (Table 1) (Golebiowska et al. 2025; Li et al. 2011).

ENS structural anomalies have also been described in idiotypic models. BTBR mice, which carry mutations at the *Itpr3* ^tf^ and *T* ^*+*^ loci, display severe abnormalities in the anterior corpus callosum, reminiscent of the reduced corpus callosum volume observed in children with ASD (Scaccabarozzi et al. 2025). In addition to core behavioral features, such as increased repetitive behavior, impaired social and communication abilities (Miller et al. 2013; Viscomi et al. 2025), these mice exhibit GI and ENS dysfunctions, manifested by delayed intestinal transit, reduced enteric neuronal density in the myenteric plexus, and increased interganglionic spacing (Table 1) (Golubeva et al. 2017).

Environmental factors also play a significant role in ASD etiology. However, relatively few environmental models have examined ENS integrity. In the maternal immune activation model, reduced levels of calcitonin gene‐related peptide (CGRP), a neuropeptide involved in modulating intestinal inflammation in the colon, have been reported (Li et al. 2023). Another model based on prenatal exposure to valproic acid, a drug used to treat epilepsy and mood disorders, shows ENS alterations characterized by reduced expression of PGP9.5, a cytoplasmic hydrolase expressed in enteric neurons. These effects are associated with an increased intestinal transit time (Table 1) (Cheng et al. 2020).

Collectively, these studies indicate that animal models of ASD, initially characterized for their behavioral impairments and brain neuronal dysfunctions, also exhibit GI dysfunctions, most notably altered gut transit. In several models, ENS structural abnormalities are observed, often reflected by changes in the number of enteric neurons. However, these alterations are not consistent across models, as some fail to show detectable ENS changes. Importantly, most studies rely on a limited set of parameters, often restricted to enteric neuron counts, thereby providing only a partial understanding of the structural and functional changes within the ENS. Future studies should aim to pursue more comprehensive approaches to better characterize ENS alterations at both structural level (e.g., synaptic connectivity) and functional level (e.g., neuronal activity and neurotransmitter release).

## 3 Gut Microbiota

### 3.1 Alteration of Gut Microbiota in ASD

The gut microbiota represents a highly diverse and complex microbial community, predominantly composed of bacteria belonging to two major phyla, Firmicutes and Bacteroides, which account for nearly 70% and 20% respectively of the total bacterial population. In addition to its role in maintaining intestinal homeostasis, increasing evidence suggests that the gut microbiota plays a significant role in modulating brain physiology and behavioral functions. In individuals with ASD, numerous studies have reported alterations in gut microbial composition compared to neurotypical controls in diverse geographical regions and populations. However, despite the growing body of literature, no single, consistent microbial signature specific to ASD has been identified. Instead, substantial inter‐study variability has been observed, likely reflecting differences in age, diet, geography, clinical heterogeneity, gastrointestinal comorbidities, sequencing methodologies, and analytical approaches (Cryan et al. 2019). However, a large number of studies converge in demonstrating significant differences in overall microbial community composition between individuals with ASD and neurotypical subjects. Among the most frequently reported alterations, an increased Firmicutes/Bacteroidetes phyla ratio has been described in several cohorts of children with ASD compared to control groups (Strati et al. 2017), although opposite results were also found (de Angelis et al. 2013). At the genus level, there was a significant increase in the relative abundance of several genera including *Dorea*, *Lachnospiraceae*, *Clostridiales*, *Collinsella*, *Akkermansia*, Clostridium, and *Lachnoclostridium* (Adams et al. 2011; Liu, Li, Wu, et al. 2019; Strati et al. 2017; Ding et al. 2020; Iglesias‐Vázquez et al. 2020; Xu et al. 2019), whereas *Parasutterella*, *Faecalibacterium*, *Paraprevotella, Bifidobacterium*, and *Bacteroides* were significantly lower in the ASD groups than in the control groups (Ding et al. 2020; Xu et al. 2019; Retuerto et al. 2024). Importantly, certain genera, such as *Erysipelotrichaceae* and *Lachnoclostridium*, have been positively correlated with ASD symptom severity, suggesting a functional link between microbial dysbiosis and clinical outcomes (Ding et al. 2020). However, a stool metagenomics study performed in children reported a weak association between gut microbiome and ASD behavioral symptoms but significant associations with a less diverse diet, stool consistency, and age (Yap et al. 2021). Prospective longitudinal studies focusing on early childhood, from 5 to 36 months of age, have revealed distinct developmental trajectories in gut microbiota composition and metabolomic profiles in infants at elevated‐likelihood of ASD (i.e., siblings of children with ASD) compared to low‐likelihood of ASD (i.e., infants without a family history of ASD) (Zuffa et al. 2023). These differences in microbiota development, concerning *Bifidobacterim*, *Clorstridium* and *Klebsiella* species, may differentially influence CNS and ENS maturation during this critical developmental window, potentially affecting behavioral and gastrointestinal functions later in life. In a subpopulation of individuals with constipation, ASD symptom severity was associated with the relative abundance of *Fusobacterium*, *Barnesiella*, *Allisonella*, *Coprobacter*, *Actinomycetaceae*, and *Olsenella* (Liu, Li, Sun, et al. 2019). The gut microbiota composition in the adult population with ASD has been less investigated than in pediatric and adolescent populations. A study conducted in adults with ASD and without intellectual disability showed a variation in the composition of the microbiota limited to the *Ruminiclostridium, Bacteroides*, and *Desulfovibrio* genera, which were more abundant in the ASD group compared to neurotypical individuals (Gonzales et al. 2021).

### 3.2 Fecal Microbiota Transfer from Patient into Mouse

While a large number of studies consistently report alterations in the gut microbiota composition in children, adolescents, and adult populations with ASD, it remains unclear whether these changes contribute to ASD‐related behavior and GI disorders. To directly address the contribution of gut microbiota to ASD symptomatology, fecal microbiota from children with ASD has been inoculated into germ‐free mice to analyze the effects on behavior and digestive functions (Xiao et al. 2021). This human fecal microbiota transfer (FMT) induced ASD‐like behavioral phenotypes characterized by increased repetitive behaviors and reduced sociability within 3 weeks post‐treatment in mice receiving fecal microbiota from ASD donors (Xiao et al. 2021). The mouse strain used for FMT, BALB/c vs. C57BL/6, appears to play an important role in the severity of the induced behavioral phenotypes, suggesting a complex interplay among genetic predispositions, gut microbiota, and behavior (Prince et al. 2025). Behavioral alterations were accompanied by a distinct microbial community structure compared to mice colonized with microbiota from neurotypical controls. In addition, significant disruptions in tryptophan and serotonin metabolic pathways as well as altered fecal concentrations of short chain fatty acids were detected in the ASD‐recipient group (Xiao et al. 2021; Prince et al. 2025). Another study demonstrated that colonization of germ‐free mice with fecal microbiota from ASD children also affected their offspring. The offspring of ASD‐colonized mice exhibited ASD‐like behavioral alterations, including deficits in sociability and increased repetitive behaviors. Consistent with these behavioral phenotypes, changes in CNS neuronal excitability were also described in the offspring (Sharon et al. 2019). This latter study suggests that perinatal factors, such as altered microbiota composition, may contribute to the etiology of autism by increasing the risk and severity of ASD‐related symptoms.

Despite these findings, the effects of microbiota transfer on the ENS remain largely unexplored. Further investigations are therefore required to elucidate the role of gut microbiota dysbiosis in ENS structure and function.

### 3.3 Fecal Microbiota Transfer as a Potential Therapeutic Tool to Treat ASD

FMT has also been explored as a potential therapeutic intervention to alleviate behavioral and GI symptoms in patients with ASD. Kang et al. performed FMT to children with ASD aged from 7 to 17 years, either by oral or rectal administration for 10 weeks, followed by an 8‐week observation period. Outcomes were compared with those of non‐treated neurotypical individuals. The study reported an approximately 80% reduction of GI symptoms at the end of treatment, including significant improvements in constipation, diarrhea, and abdominal pain. Clinical assessments further showed significant improvements in core behavioral ASD symptoms. Remarkably, both GI and behavioral benefits persisted for at least 8 weeks after the end of the treatment (Kang et al. 2017). The same group subsequently published a follow‐up study conducted on the same participants 2 years after the end of treatment. Their results showed that most of the improvements observed in GI symptoms, as well as in microbiota composition, were maintained, while behavioral symptoms had further improved. These findings suggest the long‐term safety and potential efficacy of FMT in alleviating both GI and behavioral symptoms in individuals with ASD (Kang et al. 2019). A subsequent study similarly reported that FMT in children with ASD resulted in improvements in both behavioral and GI symptoms, along with changes in serum neurotransmitters levels. The authors further observed that FMT promoted colonization by donor‐derived bacteria and shifted the bacterial community of people with ASD toward a composition more closely resembling that of typically developing controls or donors (Li et al. 2021). In cases of severe ASD, Hu et al. conducted a study involving 5 rounds of FMT during 14 weeks to a child who had shown limited response to long‐term behavioral interventions. Following FMT, both core ASD symptoms and GI disorders were significantly improved, alongside enhanced functional development (Hu et al. 2023). Furthermore, recent studies using different FMT approaches, including oral and rectal administration, have also demonstrated beneficial effects on both behavioral and GI symptoms in people with ASD (Hu et al. 2025; Varnas et al. 2025; Shirotani et al. 2026). Collectively, these studies highlight the role of the gut microbiota in regulating behavioral and GI abnormalities associated with ASD and underscore its potential as a therapeutic target for managing ASD symptoms. However, further studies involving larger patient cohorts and well‐controlled placebo groups are required to confirm these findings. Although several studies have reported beneficial effects of FMT on GI functions, to our knowledge, no study has yet analyzed the effects of FMT on ENS remodeling. Other strategies using selected specific probiotic bacteria also improved behavioral symptoms of ASD (Soleimanpour et al. 2024). Probiotics, especially strains of Lactobacillus, Bifidobacterium, and Streptococcus, have demonstrated potential in alleviating behavioral and gastrointestinal symptoms in 66% of patients (Lewandowska‐Pietruszka et al. 2023). Studies have shown that various strains of *Lactiplantibacillus plantarum* positively affect animal models of ASD and patients by enhancing gut health, improving GI function, and reducing behavioral symptoms, highlighting its therapeutic potential (Sabatini et al. 2025).

## 4 Interaction Between Gut Microbiota and ENS in Autism

The studies described above highlight the role of the gut microbiota as a potential causal factor contributing to both behavioral and gastrointestinal symptoms in ASD. Although the mechanisms through which the gut microbiota influences brain and digestive functions are not yet fully understood, they appear to involve several classes of microbiota‐derived metabolites that act on target organs by modulating a wide range of cellular processes. In the context of ASD, relatively few studies have specifically examined how a dysbiotic/altered microbiota can affect the ENS. Notably, Gonzales et al. investigated the effects of fecal supernatant isolated from the stools of adults diagnosed with ASD (Gonzales et al. 2021). Following filtration, the fecal supernatant is devoid of bacteria but contains numerous microbiota‐derived molecules, including metabolites and membrane‐associated compounds. This study demonstrated that the transfer of fecal supernatant from adults with ASD, either applied to primary ENS cultures in vitro or administered to mice in vivo, induced ENS alterations, characterized by reduced expression of glial and neuronal molecules. In particular, proteins involved in neuronal connectivity, such as βIII‐tubulin and synapsin‐1, were significantly downregulated (Gonzales et al. 2021). These findings suggest that mediators derived from dysbiotic gut microbiota from patients with ASD can induce ENS remodeling through direct or indirect pathways (Figure 1), although the specific molecules responsible were not identified in this study. In the following section, we will describe the alterations observed in various categories of bacteria‐derived metabolites, including short chain fatty acid, succinate, steroid hormones, bile acid, tryptophan derivatives, and tetrahydrobiopterin, in the context of ASD. This overview is based on evidence from both clinical studies and animal models and focuses on metabolites for which regulatory effects on the ENS have been demonstrated, and which may potentially underlie the GI symptoms observed in ASD.

### 4.1 Short Chain Fatty Acids (SCFAs)

**FIGURE 2:**
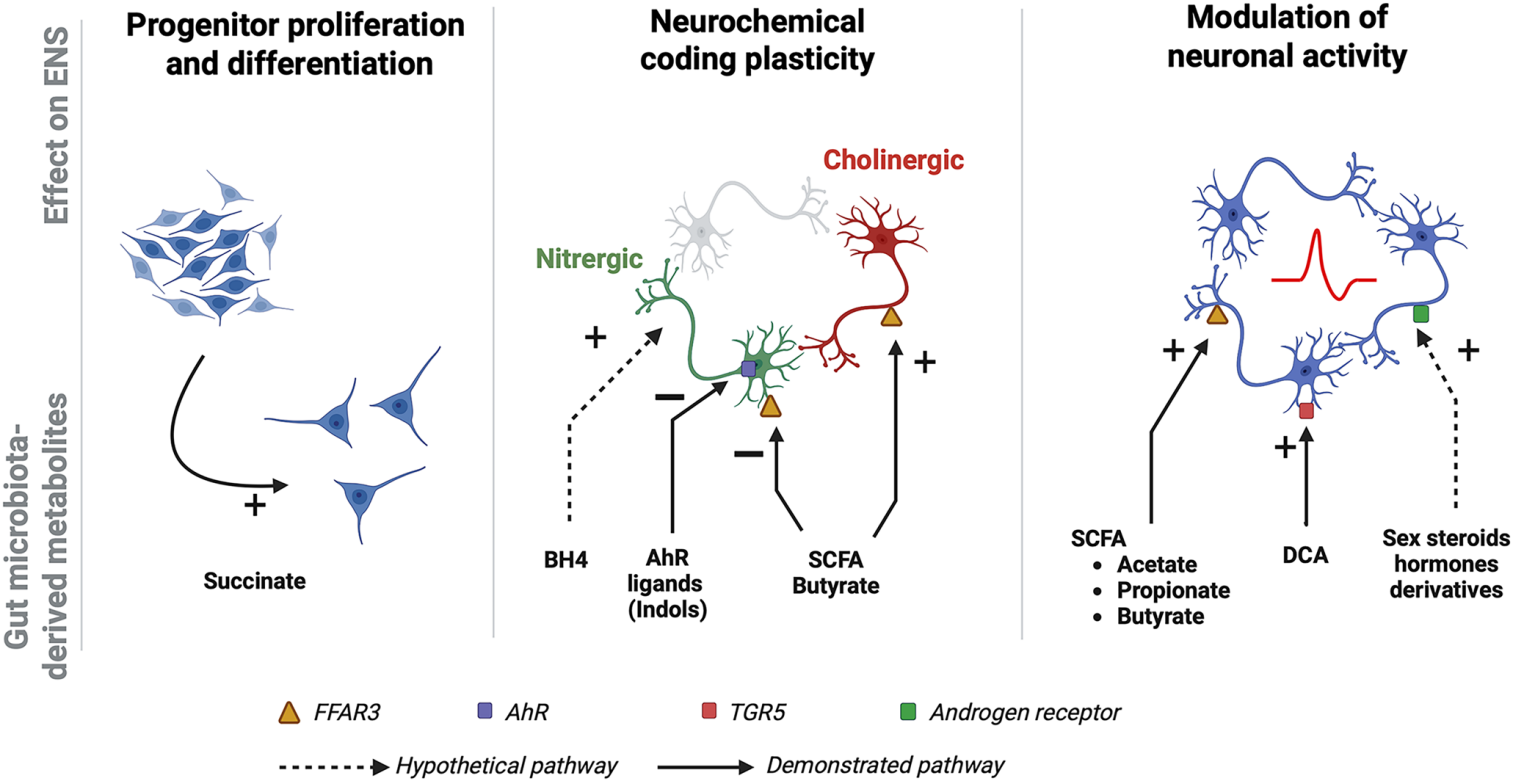
Schematic overview of gut microbiota–derived metabolite effects on the enteric nervous system development and function, which could potentially be involved in the gastrointestinal disorders associated with autism, (−) Inhibition, (+) Stimulation.

SCFAs are end products of microbial carbohydrate fermentation and reflect both gut microbiota composition and dietary fiber intake. Numerous studies have examined SCFA levels, including butyrate, propionate, acetate, and valerate, in the stools of individuals with ASD. While some studies report decreased SCFA concentrations (Adams et al. 2011; Osman et al. 2023; He et al. 2023) (Table 1), others have described increased levels (de Angelis et al. 2013; Deng et al. 2022). Overall, these findings indicate that SCFA profiles are altered in individuals with ASD, with substantial inter‐individual variability among patients. Under physiological conditions, butyrate, propionate, and acetate have been shown to play a critical role in intestinal homeostasis by serving as energy substrates for colonocytes, which in turn regulate systemic metabolism. In addition, SCFAs can modulate enteric neuron activity and gastrointestinal motility. SCFAs have been shown to influence colonic motility by decreasing the frequency of spontaneous contraction of the longitudinal muscle. This effect is abolished by tetrodotoxin, a sodium channel blocker that inhibits neuronal action potential, suggesting that SCFAs act on longitudinal muscle contractions through activation of the ENS (Ono et al. 2004). Several studies further support the role of SCFAs in modulating ENS neuronal function and neurochemical phenotype. Fung et al. demonstrated in mouse colon preparations that acetate, propionate, and butyrate can directly communicate with the ENS by acutely stimulating Ca^2+^ signaling in enteric neurons (Fung et al. 2021). Butyrate administered in vivo to young rats induced an increased percentage of cholinergic and nitregic neurons in the colon (Suply et al. 2012) while only the cholinergic neuron population was augmented in adult rats (Soret et al. 2010). Application of butyrate to primary culture of ENS increased the proportion of cholinergic neurons through a mechanism involving the monocarboxylate transporter 2 (MCT2), which is expressed in enteric neurons and facilitates the transport of SCFA across cell membranes (Soret et al. 2010). Subsequently, butyrate may promote inhibition of histone deacetylase (HDAC) activity (Davie 2003) through activation of Src family kinases, which promote acetylation of lysine 9 on histone 3 (H3K9) (Soret et al. 2010; Reddy et al. 2009). These results are consistent with the ability of butyrate to influence cell cycle and oxidative phosphorylation pathways involved in enteric neuron proliferation (Wang, Lv, et al. 2023). Additionally, butyrate has been shown to induce Ca^2+^ release from intracellular stores, leading to activation of K^+^ channels and subsequent hyperpolarization of enteric neurons (Haschke et al. 2002), and to activate IPANs by increasing their excitability (Neunlist et al. 1999). In addition to modulate histone deacetylase activity, SCFAs exert many of their effects through the activation of free fatty acid receptors, particularly free fatty acid receptor 3 (FFAR3, also known as GPR41) (Tazoe et al. 2008). FFAR3 is highly expressed in enteric neurons of both the submucosal and myenteric plexuses compared with other SCFA receptors (Figure 2) (Nøhr et al. 2013) and plays a key role in the regulation of intestinal motility. Activation of FFAR3 suppresses neural activity and chloride secretion induced by nicotinic acetylcholine receptor (nAChR) activation, and inhibits motility changes in the gut (Kaji et al. 2016; Kaji et al. 2018). In conclusion, SCFAs can directly regulate the neurochemical coding and activity of enteric neurons, thereby physiologically influencing ENS‐mediated gut motility. Altered SCFA levels reported in individuals with ASD could therefore affect enteric neuronal function and gut motility, potentially contributing to GI disorders. Further studies are still needed to better understand the role of SCFAs in GI disorders associated with ASD and to support the development of pharmacological or dietary interventions for their management.

### 4.2 Gut‐Bacteria Derived Metabolites Related to Carbohydrate Metabolism

Succinate and lactate, two specific metabolites implicated in carbohydrate metabolism, have been found to be altered in patients with ASD (Frye et al. 2024). Succinate is an intermediate molecule of the Krebs cycle, produced and metabolized in the mitochondria during the catabolism of carbohydrates, proteins, and fats. In the gut, succinate is the intermediate metabolite in the microbial fermentation of indigestible dietary and host‐derived carbohydrates into SCFAs. Several bacterial taxa, including *Bacteroides* spp., *Prevotella* spp., *Firmicutes* spp., are capable of metabolizing pentose and hexose carbohydrates to produce succinate in the intestinal tract (Wei et al. 2023). In an ASD patient cohort from the American Gut Project database, gut microbiota analysis revealed an increase in *Prevotella* spp., with succinate identified as a significant metabolite associated with the bacterial signature observed in this population (Agarwala et al. 2021). Additionally, at 36 months of age, infants at higher risk of developing ASD showed elevated fecal succinate levels compared to those at lower risk (Zuffa et al. 2023). Accordingly, other studies have reported increased plasma succinate levels in children with ASD (Sotelo‐Orozco et al. 2020). Microbiota‐derived succinate has been found to stimulate neuronal differentiation as part of a recovery mechanism following antibiotic‐induced enteric neuronal loss in vivo (Figure 2) (Aydin et al. 2024). However, it remains to be determined whether succinate mediates neuronal recovery through a direct action on ENS precursors or indirectly via interaction with other gut‐resident cell types (Aydin et al. 2024). One possibility is that elevated succinate levels in individuals with ASD may influence enteric neuronal homeostasis by increasing neuronal differentiation, thereby contributing to disturbances in gut physiological homeostasis. Lactate is a biomarker of mitochondrial dysfunction, indicating abnormalities in mitochondrial carbohydrate metabolism (Frye et al. 2024). Elevated cerebral and blood lactate levels were shown in ASD patients (Frye et al. 2024; Maier et al. 2023). In the gut, elevated lactate‐producing bacteria such as *lactobacillus* have been reported to be elevated in the feces of ASD patients (Xu et al. 2019; Tomova et al. 2015). The lactate‐producing bacteria can express glutamic acid decarboxylase (GAD), which converts glutamate into GABA, a neurotransmitter of both CNS and ENS, thereby promoting its accumulation notably in the mouse small intestine (Wang et al. 2025; Zhong et al. 2023). Lactate is also an important energy substrate for neurons and acts as a signaling molecule regulating neuronal excitability (Cauli et al. 2023). Elevated lactate levels reported in ASD may affect metabolic signaling and excitability in enteric neurons.

### 4.3 Steroid Hormones

More than 1000 varieties of naturally occurring steroids have been identified, including endogenous sex steroid hormones (androgens, estrogens, and progestogens) and corticosteroids, produced by the gonads and adrenal glands, respectively. Given the sex differences in ASD prevalence, with a male‐to‐female ratio of about 4:1, potential links between sex hormone levels and ASD have been investigated. A recent meta‐analysis indicated elevated serum and fecal androgen levels, such as free testosterone and dihydrotestosterone, in individuals with ASD compared to age‐ and sex‐matched controls (Wang et al. 2024). In addition, allopregnanolone, a metabolite of progesterone, showed reduced levels in the serum of adult males with ASD (Chew et al. 2021). Similarly, increased steroid hormone biosynthesis has been observed in a valproic acid‐induced rat model of ASD, compared to control rats (Gu et al. 2022). Interestingly, the level of steroid hormones can be influenced by the gut microbiota, as several bacterial species express enzymes capable of metabolizing steroid hormones. For instance, several bacterial species produce β‐glucuronidase (GUS), an enzyme which converts inactive estrogens back into their active forms. GUS enzymes are also involved in the deglucuronidation of dihydrotestosterone (DHT) and testosterone, thereby activating these hormones (Diviccaro et al. 2025; Vítků and Hampl 2023). In addition, certain gut bacteria are able to produce derivatives of sex steroid hormones, such as allopregnanolone, a metabolite of progesterone, as well as progestins, by converting glucocorticoids present in the bile through 21‐dehydroxylation (McCurry et al. 2024). Similarly, other bacteria, such as *Clostridium scindens* , express steroid‐17,20‐desmolase, an enzyme that converts glucocorticoids into 11β‐hydroxyandrostenedione, a precursor of androgens (Ridlon et al. 2013). Consistently, antibiotic‐induced microbial depletion in mice leads to decreased serum testosterone levels, suggesting that androgen levels depend in part on microbial activity (Lagomarsino et al. 2026). Sex steroid hormones act beyond sexual differentiation and reproduction, as they are involved in multiple physiological systems, including the gut and the CNS. Interestingly, androgen receptor (AR) expression has been identified in enteric neurons, and AR signaling plays a crucial role in maintaining normal colonic motility in adult mice (Figure 2) (Rastelli et al. 2022). In particular, microbial depletion in mice has been shown to decrease serum testosterone levels and abolish AR expression in enteric neurons, resulting in colonic motor dysmotility (Lagomarsino et al. 2026). Interestingly, the majority of AR‐positive enteric neurons are nitrergic neurons, expressing neuronal nitric oxide synthase, while cholinergic enteric neurons do not express AR, suggesting that androgen signaling may be restricted to the inhibitory component of the peristaltic reflex circuit (Lagomarsino et al. 2026). These findings suggest that, in the context of ASD, elevated serum levels of androgenic molecules, such as testosterone or dihydrotestosterone, could specifically affect ENS nitrergic signaling, potentially leading to colonic dysmotility. Moreover, sex steroid hormones can modulate neurotransmitter‐mediated signaling in neurons. For instance, allopregnanolone acts as a modulator of GABA‐A receptors, which are expressed in both the CNS and ENS and are involved in regulating colonic contractions (Diviccaro et al. 2025; Bäckström et al. 2022; Seifi et al. 2014). In ASD condition, reduced serum allopregnanolone levels may alter GABA‐A receptor signaling in enteric neurons, then potentially affecting colonic contractility.

### 4.4 Bile Acid (BAs)

Primary bile acids (BAs), composed of cholic acid (CA) and chenodeoxycholic acid (CDCA), are synthesized from cholesterol in the liver and are secreted in the intestine by the gallbladder to facilitate lipid digestion and absorption (Darmanto et al. 2025). Primary BAs also serve as substrates to produce secondary BAs by the gut microbiota (Hurley et al. 2022). The major secondary BAs, deoxycholic acid (DCA) and lithocholic acid (LCA), are produced by the gut microbiota through sequential microbial enzymatic reactions involving deconjugation (i.e., removal of glycine or taurine moieties) followed by 7α‐dehydroxylation of CA and CDCA, respectively. Elevated fecal levels of DCA have been reported in individuals with ASD compared to controls (Gonzales et al. 2021). In addition, secondary BA profiles were found to be dysregulated in the BTBR T+ Itpr3tf/J mouse strain, an idiopathic model of ASD (Golubeva et al. 2017). In this model, a reduced abundance of gut bacterial taxa capable of producing secondary BAs, such as *Bifidobacterium* and *Blautia*, has been observed. These microbial and metabolic alterations are associated with GI disorders and changes in the ENS, characterized by a decreased number of enteric neurons in the myenteric plexus, particularly within the nitrergic neuronal population. Thus, variations in secondary BA levels resulting from imbalances in bacterial production or host metabolism may contribute to digestive disorders and ENS abnormalities in the context of ASD. Studies in mice have shown that DCA administration increased gut motility (Li et al. 2021; Alemi et al. 2013; Shiff et al. 1982) and permeability (Sun 2004), through a mechanism involving the ENS. Accordingly, DCA has been shown to directly modulate enteric neuronal activity through interacting with the bile acid receptor TGR5 (Figure 2) (Le Dréan et al. 2026), which is expressed by enteric neurons (Poole et al. 2010).

### 4.5 Tryptophan Metabolism

In the body, tryptophan is primarily metabolized through three major pathways: the indole derivative pathway, the kynurenine pathway, which accounts for more than 90% of dietary tryptophan metabolism, and the serotonin (5‐hydroxytryptamine, 5‐HT) pathway. In the gut, approximately 5% of dietary tryptophan is metabolized through the indole pathway by the gut microbiota (Agus et al. 2018). Indole derivatives such as indole‐3‐pyruvate (IPYA), indole‐3‐aldehyde (IAld), indole‐3‐acid‐acetic (IAA), indole‐3‐propionic acid (IPA), indole‐3‐acetaldehyde (IAAld), and indoleacrylic acid are known ligands for the aryl hydrocarbon receptor (AhR). Tryptophan metabolism is increasingly recognized as an important contributor to ASD pathophysiology. A recent study demonstrated that fecal levels of specific tryptophan‐related metabolites, including kynurenate, were significantly lower in patients with ASD and were associated with altered activity in the insular and cingulate cortices, brain regions previously implicated in ASD severity and symptomatology (Aziz‐Zadeh et al. 2025). At the intestinal level, the gut bacteria *Lactobacillus* spp., also known to be producers of AhR ligands, have been reported to be elevated in the feces of ASD patients (Xu et al. 2019; Tomova et al. 2015). AhR signaling has a role in maintaining intestinal homeostasis, including intestinal barrier renewal and integrity as well as regulation of different immune cell types. Enteric neurons express AhR, which has been shown to induce enteric neuronal toxicity by reducing the number of nitrergic neurons (Figure 2), leading to prolonged intestinal transit time in mice (Vijay et al. 2023). Regarding the kynurenine pathway, the gut microbiota is a key player in stimulating indoleamine 2,3‐dioxygenase 1 (IDO1) activity, which catalyzes the conversion of tryptophan to kynurenine. Bacteria from the *Pseudomonas* genus, which are reduced in ASD patients, are capable of producing kynurenine from tryptophan (Vujkovic‐Cvijin et al. 2013; Yang et al. 2024). Kynurenine pathway end‐products are involved in the regulation of neurotransmission, inflammation, and immune response (Agus et al. 2018). Notably, kynurenine acid is an endogenous antagonist of NMDA receptors and may contribute to gastrointestinal neuroprotection against inflammation and glutamate‐induced neurotoxicity (Kaszaki et al. 2012). The gut microbiota also plays a major role in intestinal 5‐HT production (Yano et al. 2015). *Escherichia coli* , which is found at lower levels in ASD patients, is capable of synthesizing 5‐HT (Yano et al. 2015). More broadly, gut microbiota modulates 5‐HT production through the release of SCFAs, which stimulate the expression of tryptophan hydroxylase 1 (TpH1) in enterochromaffin cells, thereby promoting 5‐HT synthesis (Yano et al. 2015). In addition, bacterial tryptophan catabolites such as indole and its derivatives can activate the Trpa1 receptor on enteroendocrine cells of the intestinal epithelial barrier, triggering 5‐HT secretion and rapid activation of enteric and vagal neurons (Ye et al. 2021).

### 4.6 Tetrahydrobiopterin (BH4) Metabolism Pathway

Tetrahydrobiopterin (BH4) is an essential enzymatic cofactor involved in the synthesis of monoamine neurotransmitters, the metabolism of phenylalanine and lipid esters, and the production of nitric oxide (NO) (Eichwald et al. 2023). BH4 promotes and stabilizes nitric oxide synthase (NOS) dimerization, protects the enzyme from proteolysis, and increases the binding of the substrate L‐arginine to NOS (Eichwald et al. 2023). Reduced levels of BH4 have been detected in the blood, urine, and cerebrospinal fluid of children with ASD (Eto et al. 1992) as well as in the feces of an ASD mouse model (Cntnpa2−/−) (Buffington et al. 2021). Interestingly, BH4 can also be produced by the gut microbiota, particularly by members of the A*ctinobacteria* phylum (Belik et al. 2017), which has been reported to be decreased in children with ASD (Belik et al. 2017), although other studies have found the opposite trend (Iglesias‐Vázquez et al. 2020). Regarding the ENS, administration of BH4 in diabetic mice has been shown to restore the ENS nitrergic pathway by increasing neuronal NOS expression and enhancing nitrergic‐induced gastric muscle relaxation (Figure 2) (Gangula et al. 2010). These findings suggest a potential implication of BH4 in the regulation of nitrergic enteric neurons, which may contribute to the pathophysiology of ASD.

## 5 Conclusion and Future Directions

In this review, we summarize the alterations in the gut microbiota and the ENS reported in autism, in both patients and animal models. We provide an overview of microbiota‐derived metabolites associated with ASD that have been shown to exert regulatory functions on the ENS, including short‐chain fatty acids, succinate, steroid hormones, bile acids, tryptophan derivatives, and tetrahydrobiopterin. This review highlights bacterial metabolite–ENS communication as a key mechanism leading to ENS remodeling and potentially underlying gastrointestinal (GI) symptoms in ASD. Further investigations are required to achieve a more comprehensive characterization of microbiota‐derived metabolites relevant to ASD in both animal models and humans. Such progress would enhance our understanding of the pathophysiological mechanisms involved in ASD and could also provide promising biomarkers and potential targets for novel therapeutic strategies. Elucidating the selective effects of different classes of bacterial metabolites on the ENS could lead to the development of combinatorial approaches that integrate several metabolites with complementary functions to modulate ENS activity and alleviate GI symptoms. In addition to bacterial metabolites, other microbiota‐derived compounds, including membrane components, flagellar fragments, and extracellular vesicles, also represent promising candidates for mediating gut microbiota–ENS communication. Overall, this emerging field opens new perspectives for investigating the interactions between the gut microbiota and the ENS in the context of ASD. Further studies are warranted to improve our understanding of microbiota–ENS interactions in ASD.

## Funding

This work was supported by Agence Nationale de la Recherche (ANR‐22‐CE14‐0043, ANR‐19‐CE14‐0024), ECOS‐Sud (C23S01), Conseil Régional des Pays de la Loire (R20056NN, R23066NN), FHU Exac.t, Fondo Nacional de Desarrollo Científico y Tecnológico (#1250762, #230036), and ANID Becas Doctorado National (21211419).

## Conflicts of Interest

The authors declare no conflicts of interest.

## Data Availability

The data that support the findings of this study are available on request from the corresponding author. The data are not publicly available due to privacy or ethical restrictions.
